# Selenium in Human Health and Gut Microflora: Bioavailability of Selenocompounds and Relationship With Diseases

**DOI:** 10.3389/fnut.2021.685317

**Published:** 2021-06-04

**Authors:** Rannapaula Lawrynhuk Urbano Ferreira, Karine Cavalcanti Maurício Sena-Evangelista, Eduardo Pereira de Azevedo, Francisco Irochima Pinheiro, Ricardo Ney Cobucci, Lucia Fatima Campos Pedrosa

**Affiliations:** ^1^Postgraduate Program in Nutrition, Federal University of Rio Grande do Norte, Natal, Brazil; ^2^Department of Nutrition, Federal University of Rio Grande do Norte, Natal, Brazil; ^3^Graduate Program of Biotechnology, Laureate International Universities - Universidade Potiguar, Natal, Brazil; ^4^Medical School, Laureate International Universities - Universidade Potiguar, Natal, Brazil

**Keywords:** selenium, gut microbiota, selenocompounds, selenoproteins, selenium metabolism

## Abstract

This review covers current knowledge of selenium in the dietary intake, its bioavailability, metabolism, functions, biomarkers, supplementation and toxicity, as well as its relationship with diseases and gut microbiota specifically on the symbiotic relationship between gut microflora and selenium status. Selenium is essential for the maintenance of the immune system, conversion of thyroid hormones, protection against the harmful action of heavy metals and xenobiotics as well as for the reduction of the risk of chronic diseases. Selenium is able to balance the microbial flora avoiding health damage associated with dysbiosis. Experimental studies have shown that inorganic and organic selenocompounds are metabolized to selenomethionine and incorporated by bacteria from the gut microflora, therefore highlighting their role in improving the bioavailability of selenocompounds. Dietary selenium can affect the gut microbial colonization, which in turn influences the host's selenium status and expression of selenoproteoma. Selenium deficiency may result in a phenotype of gut microbiota that is more susceptible to cancer, thyroid dysfunctions, inflammatory bowel disease, and cardiovascular disorders. Although the host and gut microbiota benefit each other from their symbiotic relationship, they may become competitors if the supply of micronutrients is limited. Intestinal bacteria can remove selenium from the host resulting in two to three times lower levels of host's selenoproteins under selenium-limiting conditions. There are still gaps in whether these consequences are unfavorable to humans and animals or whether the daily intake of selenium is also adapted to meet the needs of the bacteria.

## Selenium Forms, Food Sources, and Bioavailability

The organic forms of Se are found as a sulfur amino acid analog, selenomethionine (SeMet), selenocysteine (SeCys), and as methylated derivatives. The inorganic forms correspond to Se salts such as selenate (Se${\text{O}}_{4}^{-2}$) and selenite (Se${\text{O}}_{3}^{-2}$) (1). SeMet is found in plant- and animal-origin products as well as in some food supplements (2). On the other hand, SeCys is found primarily in animal-derived food (3), whereas selenium-methylselenocysteine (SeMeCys) is a natural monomethylated organic Se found in some vegetables such as garlic, onion, broccoli, and leeks (2–4). Among the inorganic forms, selenite is present mainly in food supplements, while selenate is found in plant and fish sources (3). These forms of Se have been used to biofortify some vegetables (5, 6).

**Figure 1:**
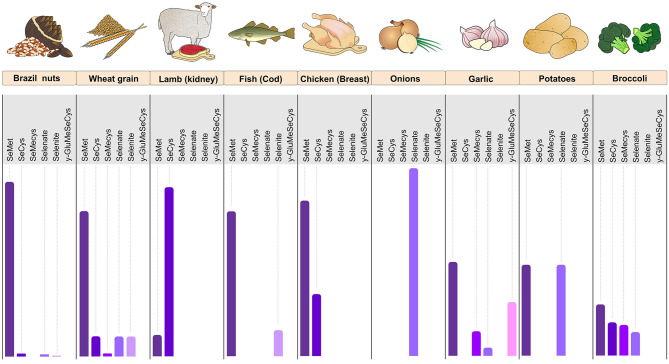
Foods rich in Se with their relative proportions of SeMet, SeCys, SeMeCys, selenate, selenite, and γ-GluMeSeCys. The figure shows the predominance of SeMet in Brazil nuts, wheat grain, fish cod, and chicken (breast). Lamb meat (kidney) is rich in Se mainly in the SeCys form, whereas onion has Se almost exclusively in the form of selenate. Garlic, potato, and broccoli have a balanced proportion of the various forms of Se. Se, selenium; SeMet, selenomethionine; SeCys, selenocysteine; SeMeCys, selenium-methylselenocysteine; γ-GluMeSeCys, γ-glutamyl-Se-methyl-selenocysteine (Figure illustration by Francisco Irochima Pinheiro).

**Figure 2:**
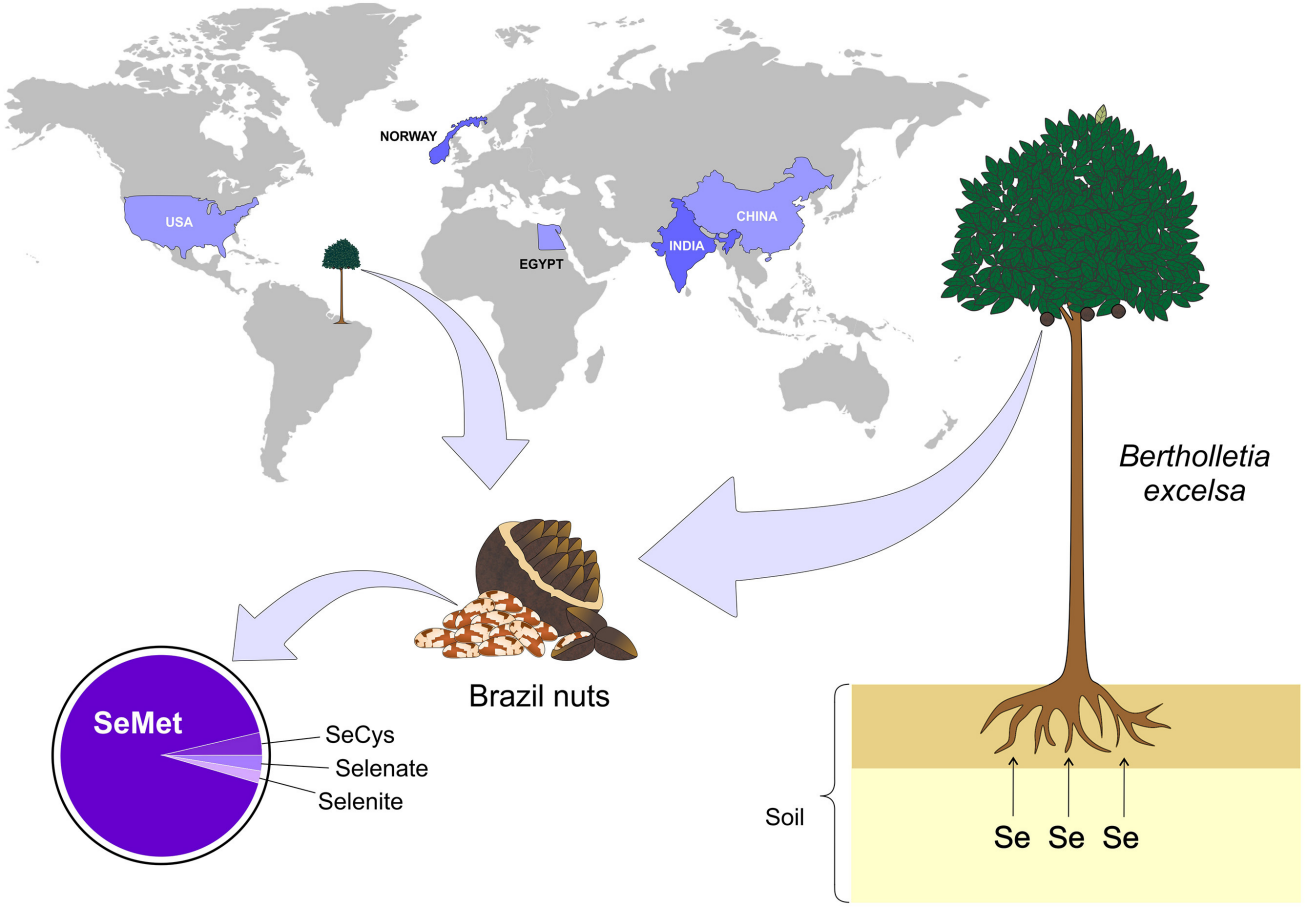
Main countries with documented Se-rich soils. The presence of Se-rich soil is not uniform in the world, but some countries such as Egypt, China, India, Norway, and USA stand out among the others. On the other hand, some plants are more capable of retaining Se, for example - *Bertholletia excelsa* (Brazil nuts), which is a typical tree of the northern region of Brazil. Se, selenium; SeMet, selenomethionine; SeCys, selenocysteine (Figure illustration by Francisco Irochima Pinheiro).

Brazil nuts, cereals, meat, fish, seafood, milk, and nuts are the best sources of Se (7) (Figure 1). The interaction of fish and seafood with mercury results in insoluble Se derivatives that can reduce its bioavailability (8). In fact, the bioavailability of Se depends primarily on its chemical form. In general, the organic forms are more quickly absorbed and are usually used for the biosynthesis of selenoproteins (9). In addition, the amount of protein, fat, and heavy metal in the diet influence the bioavailability of Se (7–10). High levels of Se are present in some herbal plants such as *Astragalus bisulcatus* and *Brassicaceae* (broccoli) (11). *Bertholletia excelsa*, known as Brazil nut, is one of the highest sources of Se with concentrations that range from 1.80 to 320.80 μg Se/g (12). In addition, the content of Se in the soil has a major influence on the amount of this metal in food, being related to its deficiency and toxicity in some regions. The Se content in the soil usually ranges from 1 to 1.5 μg Se/g, reaching 5.0 μg Se/g in seleniferous soils (13) (Figure 2).

## Selenium Absorption, Metabolism, Excretion, and Biomedical Applications

**Figure 3:**
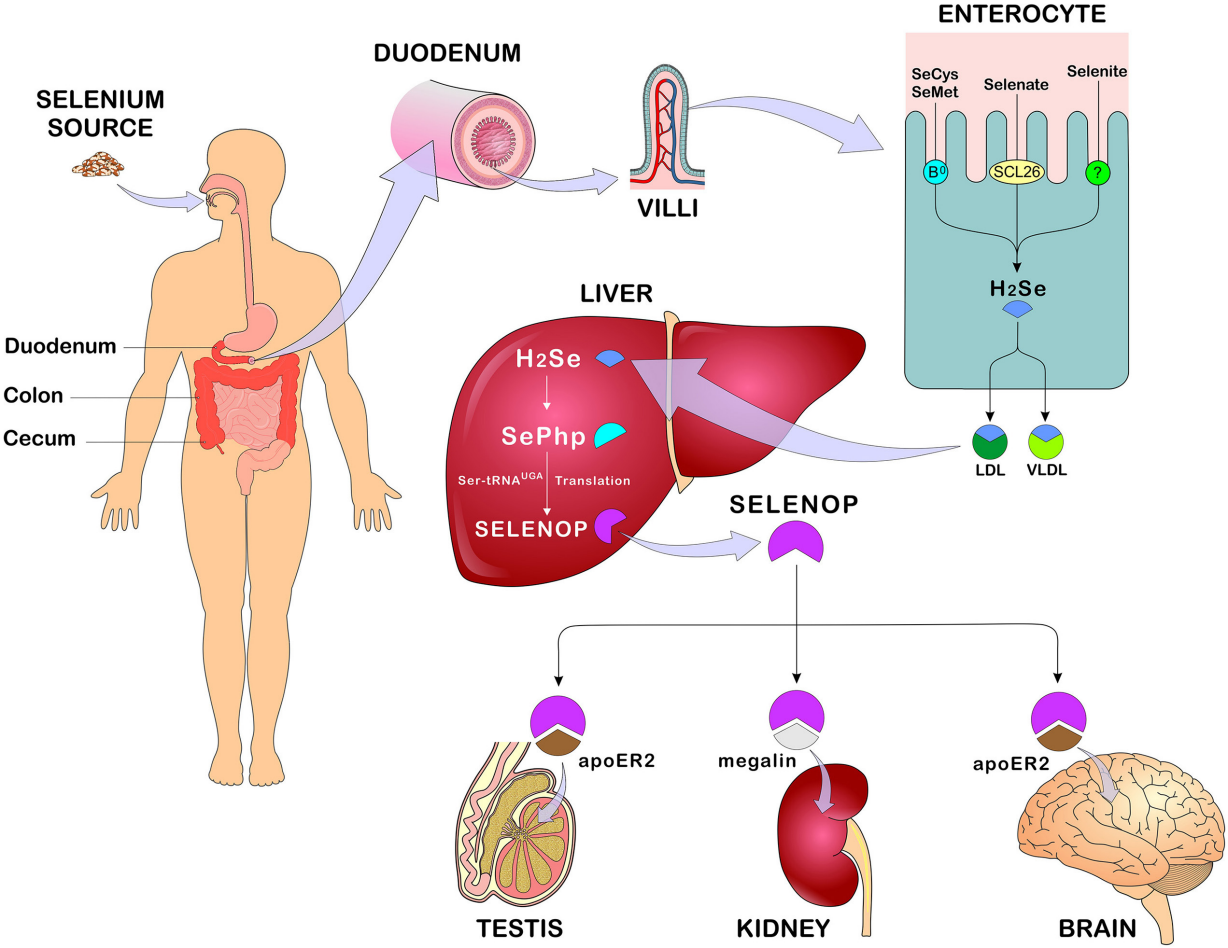
Se absorption, metabolism, and distribution. After eating Se-rich foods in its organic and/or inorganic form, Se absorption occurs in the duodenum, cecum, and colon. In enterocytes, SeMet and SeCys are absorbed by active transport (systems B0 and b0 + rBAT) while selenate is absorbed by passive transport (anion changers of the SLC26 gene family). After absorption, all forms of Se are converted to H_2_Se through reactions that occur in the enterocyte and transported in the blood bound LDL, VLDL (mainly). In the liver, H_2_Se is converted to SePhp and incorporated into selenoproteins in the form of SeCys. Transport to other tissues such as testis, kidneys, and brain occurs mainly in the form of SELENOP through receptor-mediated endocytosis - apoE2 and megaline. Se, selenium; SeMet, selenomethionine; SeCys, selenocysteine; H_2_Se, hydrogen selenide; LDL, low-density lipoprotein; VLDL, Very low-density lipoprotein; SePhp, selenophosphate; SELENOP, selenoprotein P; apoE2, apolipoprotein E receptor 2 (Figure illustration by Francisco Irochima Pinheiro).

Dietary Se intake from either organic or inorganic origin is absorbed in the gastrointestinal tract and subsequently transported to the liver, where it is metabolized and used for producing selenoproteins, followed by its distribution to other tissues of the body. Selenoamino acids are actively transported in the duodenum, cecum, and colon through various membrane transport mechanisms, whereas selenate is transported by anion exchangers from the family of the SLC26 gene. On the other hand, there is insufficient evidence on the transport of the other forms of Se (14). Se absorption, metabolism and body distribution are represented in Figure 3.

Although the metabolic route differs depending of the Se source, all the absorbed Se is converted to hydrogen selenide (H_2_Se) in the enterocytes before the specific incorporation of selenocysteine takes place in the active site of the selenoproteins (15). SeMet undergoes transulfurization reactions, in which cystathionine beta-synthetase catalyzes the formation of selenocystathionin, being further converted to SeCys by cystathionine gamma-lyase followed by conversion to H_2_Se by selenocysteine lyase. SeCys, from both food and the SeMet pathway, will also be reduced to H_2_Se (16, 17). Alternatively, SeMet can also be incorporated non-specifically into proteins, such as albumin and hemoglobin, replacing methionine (3). As for the inorganic forms, selenate is converted to selenite followed by reduction to H_2_Se by thioredoxin reductase (TXNRD) and thioredoxin, as well as by glutathione to form selenodiglutathione (GS-Se-SG). Glutathione reductase converts the latter to glutathioselenol (GS-SeH) which reacts with glutathione to form H_2_Se (3, 18–20). On the other hand, SeMeCys and the synthetic Se derivatives selenobetaine, methylseleninic acid, and methylselenocyanate are converted into methylselenol (CH_3_)SeH through the enzyme cystathione gamma-lyase, followed by demethylation to become H_2_Se (21, 22).

In view of this cascade of reactions, all H_2_Se regardless of its origin will be transported in the blood linked to VLDL and LDL fractions as well as to other proteins (albumin and alpha-globulin). In the liver, H_2_Se is converted to selenophosphate (SePhp) via selenophosphate synthetase (SEPHS), which will be incorporated into selenoproteins in the form of SeCys. For selenocysteine synthesis, the UGA codon (TGA) is used as an initiation codon, requiring a specialized tRNA (ribonucleic acid carrier), which, after several reactions from the seryl-tRNA, provides information in a targeted manner to the ribosomes that translate mRNAs (messenger ribonucleic acid) to selenoproteins (3, 18–20). Se is transported to tissues such as brain, kidneys, and testicles, mainly in the form of selenoprotein P (SELENOP) through endocytosis mediated by apolipoprotein E receptor 2 (apoE2) and megaline (14).

H_2_Se can also be methylated by thiol-S-methyltransferase before being excreted. The main form of Se excretion is through urine, however, in cases of excessive consumption, respiratory excretion might occur. Excretion by the lungs occurs when the elimination of Se in the form of trimethyl selenonium (CH_3_)_3_Se in the urine becomes saturated, whose elimination occurs mainly in the form of volatile dimethyl selenide (CH_3_)_2_Se (23). In situations of moderate consumption of Se, the main monomethylated compound eliminated through kidneys is a seleno sugar namely 1β-methylseleneN-acetyl-D-galactosamine. The non-absorbed Se from food is incorporated into the bile, pancreatic, and intestinal secretions, being eliminated in the feces (23).

Se functionality occurs in the form of selenoproteins that are encoded by the insertion of SeCys by the UGA codon in mRNA under specific conditions. Most of these selenoproteins are involved in the regulation of redox signaling and are grouped into families such as glutathione peroxidases (GPXs), iodothyronine deiodinases (DIOs), TXNRDs, and SELENOP. Thus, the main biomedical applications attributed to Se are related to its antioxidant activity, regulation of thyroid hormone metabolism, anticarcinogenic property, and prevention of cardiovascular diseases. SELENOP acts as the main Se transporter for peripheral tissues in addition to performing extracellular antioxidant function (14, 22).

## Selenium Biomarkers

**Figure 4:**
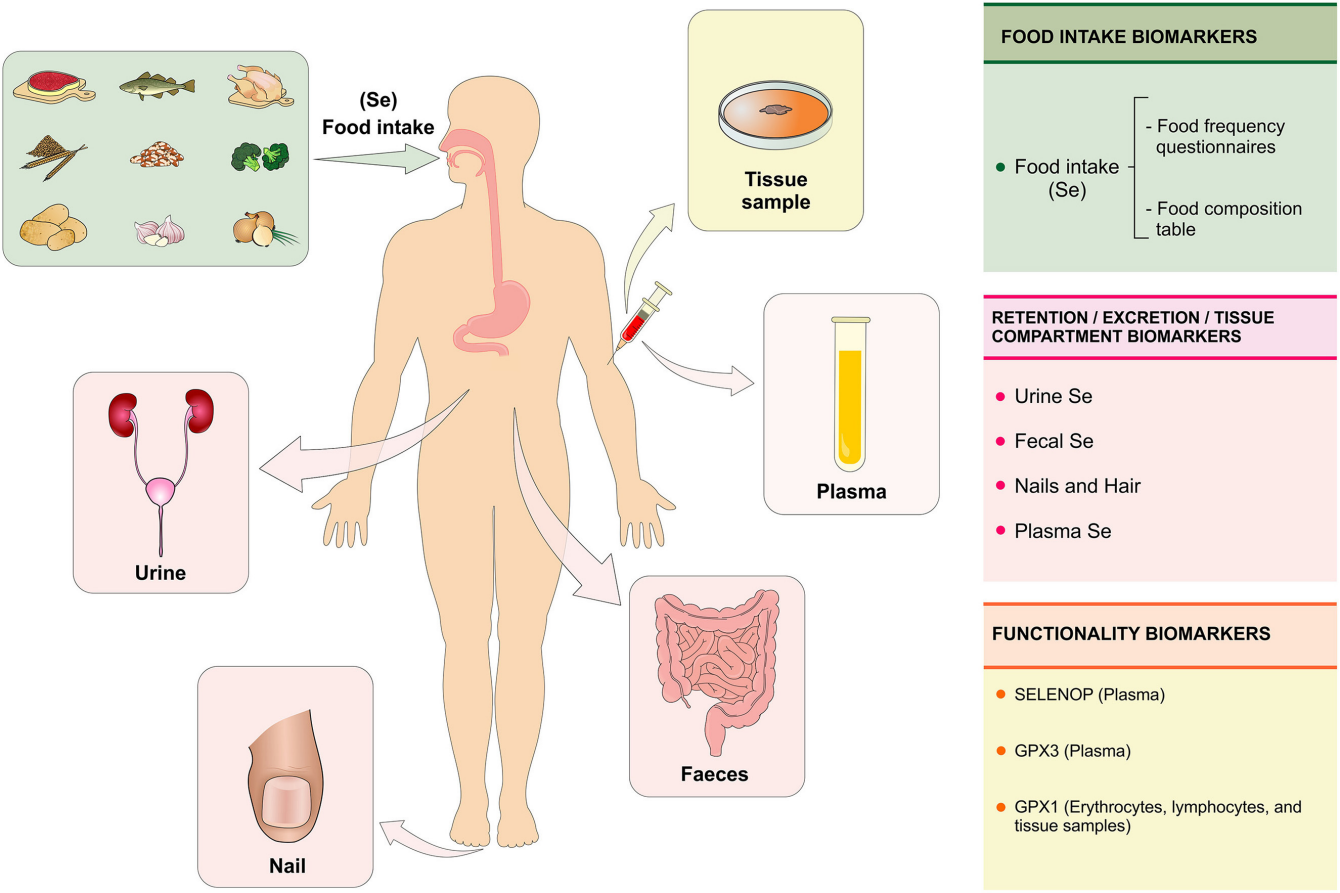
Se biomarkers. The Se status is evaluated with the purpose of quantifying the biologically active or potentially active nutrient as a function of Se intake, retention, and metabolism in the body. The Se status can be assessed at three levels using specific biomarkers: (1) biomarkers of intake (assessment of food consumption through the food frequency questionnaire); (2) biomarkers of retention/excretion and concentration in tissues (urine, feces, nails, hair, and plasma) and (3) biomarkers of selenium functionality (SELENOP and GPX3 in plasma and GPX1 in erythrocytes, lymphocytes, and tissue samples). Se, selenium; SELENOP, selenoprotein P; GPX1, glutationa peroxidase; GPX3, glutationa peroxidase 3 (Figure illustration by Francisco Irochima Pinheiro).

The evaluation of Se status determines the amount of this biologically active nutrient as a function of intake, retention, and metabolism. Thus, the Se status can be assessed at three levels using biomarkers of intake, retention/excretion, and concentration on tissues as well as biomarkers of functionality (22) (Figure 4).

### Biomarkers of Intake

The assessment of Se intake can be performed using methods of assessing food consumption, such as the food frequency questionnaire. The Se content on foods is estimated using food composition tables (22). Algeria 's population consumes a wide variety of Se-rich foods, such as seafood, meat, eggs, milk, and legumes, however, no significant associations were found between dietary patterns and Se biomarkers, such as Se in plasma, SELENOP, and GPX. Predicting the Se status from food consumption remains a challenge due to the lack of precision of nutrient content in food composition tables, considering the variation in the Se concentration of foods as a function of Se content in the soil (22, 24). In addition, Se from the diet affects colonization of the microbial intestine, which in turn influences the host's Se status and selenoproteoma expression (25).

### Biomarkers of Retention/Excretion and Se Concentrations in Tissues

The retention of Se in the body can be assessed by the difference between the amount of Se ingested and the sum of Se in the urine and feces, which requires the collection of total urine and excreted feces for a few days. Alternatively, it is recommended to evaluate the concentration of creatinine in the urine to reduce the error associated with the variation in urinary excretion. Renal excretion is the main route of elimination of absorbed Se (22). Genetic and environmental factors, as well as body size, age, and sex can influence the retention and excretion of Se in the urine (26). However, studies on Se in urine for biological monitoring are scarce, especially with regard to occupational exposure, in which inhalation is the main route of exposure. After inhalation of high concentrations of Se by workers, inflammatory effects were observed in the respiratory tract (27). An increased intake of Se is reflected rapidly in the increased excretion of Se in the urine (28). The evaluation of Se in urine can be a sensitive parameter for occupational exposures of Se in the short term, but the knowledge about specificity and kinetics of this elimination pathway is still little explored (27).

The measurement of the Se concentration in urine is considered as a potentially viable biomarker of Se status in population studies. Additionally, the concentration of Se in the urine can be used to identify regional variations in the status of Se and might reflect differences in the amount of Se in food according to the type of soil. This evidence supports the need for reviewing the policies of national systems for monitoring micronutrient deficiencies including Se (26).

The concentration of Se in the nail is considered a superior biomarker of Se status, as it provides an integrated measurement of long term exposure (up to 1 year), while blood biomarkers indicate a short term exposure (29). Toenails are considered non-invasive matrices and are used in large epidemiological studies because they present slow growth, easy collection and have less influence from external contamination. The standardization of sample collection, quality control, and analytical techniques are important to consolidate the usefulness of this matrix in epidemiological studies (30). The Se content in nails has a direct relationship with SELENOP and most organic forms of Se, especially SeCys, whereas it has an inverse relationship with the amount of the inorganic forms, such as selenite and selenate. This opposite behavior may be related to the composition of human nails, which are mainly made of proteins rich in cysteine, the latter being able to form complex with Se (31).

There are controversies about the use of Se content in nails and hair as a way to assess the effectiveness of Se supplementation. A systematic review performed with 18 Se supplementation studies found no evidence to support the use of the Se content in the nail and hair as a reliable measurement of effectiveness of Se supplementation (32). Se content in hair has been used to assess long-term Se status in epidemiological studies, offering the advantage of being a low-cost method and easy to store the samples. The Se concentration in hair and nails are excretory forms of Se. Therefore, both reflect the previous status, being more useful as biomarkers in studies of populations with stable dietary patterns (22).

Plasma Se concentration is a more useful biomarker to assess Se status in humans, considering the stability of Se in this compartment (22). A systematic review has recommended the use of plasma Se concentration as a reliable biomarker in supplementation studies with adults of both sexes. The measurement of Se in plasma has shown to be effective in reflecting changes in the amount intake (supplementation) in individuals with intermediate or high Se concentrations at baseline. In addition, this review highlights the usefulness of Se in erythrocytes and whole blood as markers of Se status, both of which are reported as markers of long-term status (32).

### Biomarkers of Selenium Functionality

Biomarkers of Se functionality include SELENOP, which comprises 20–70% of Se in plasma; GPX3, which comprises 10 to 25% of Se in plasma and GPX1, which can be tested on erythrocytes, lymphocytes, oral cells, and tissue biopsy specimens (22). Plasma SELENOP has been considered a useful biomarker of Se status in populations with relatively low Se intake, but not in populations with high intake that already had high levels of Se before supplementation began (32). SELENOP has shown to be a reliable and sensitive Se status biomarker, providing dose response that can be used to estimate the Se intake required to reach its plateau in the plasma (33). It seems that SELENOP reaches a plateau after supplementation with selenite at doses around 400 μg/day (34).

GPX is one of the main selenoproteins that belongs to the cellular antioxidant defense system. The recommended Se intake was calculated based on optimal plasma GPX3 activity due to the hierarchy of selenoproteins. It also considers the necessary amounts of Se for normal concentrations of other biologically Se compounds (35). A cohort study conducted with 51 participants with adequate Se intake investigated the association between plasma Se, GPX activity, and SELENOP. The results were discrepant between plasma Se concentrations and GPX activity, suggesting other factors may impact the activity of this enzyme such as genetic polymorphisms (36, 37).

## Selenium and Diseases

Se plays a crucial role in normal physiology and contributes to the pathophysiology of various diseases. Due to its antioxidant and anti-inflammatory properties, several studies have evaluated the impact of Se status in conditions characterized by inflammation and oxidative stress, which includes diabetes, metabolic syndrome, cancer, cardiovascular, and neurodegenerative diseases (38).

Inadequate serum Se levels may increase the risk for the development of several diseases, especially cardiovascular disorders, but it also may lead to cancer, liver diseases, and arthropathies. On the other hand, excessive consumption of Se can cause selenosis, which leads to symptoms such as fatigue, tachycardia, nausea, and diarrhea. Chronic selenosis can cause liver and kidney necrosis, neurological disorders and might compromise the reproductive and immune systems (39).

In three large cohorts, the high serum Se concentration was associated with reduced mortality (40). Another larger study, in which more than 13,000 adults were followed for 12 years, revealed that serum Se greater than or equal to 135 μg/L were associated with reduced cancer mortality (41, 42). Meta-analysis involving 16 prospective studies demonstrated an inverse relationship between Se status and cardiovascular risk (43). Likewise, a systematic review with meta-analysis involving 13 studies revealed that high physiological levels of Se are associated with lower incidence and lower mortality from cardiovascular disease (CVD) (44). In another meta-analysis in which more than 40 thousand participants in randomized clinical trials were included, the authors found that Se supplementation decreases the serum levels of C-reactive protein and increases the levels of GPX, suggesting a positive effect on reduction of inflammation and oxidative stress in cardiovascular diseases (45).

Selenium-binding protein 1 (SELENBP1), an intracellular protein involved in Se metabolism and redox control, has been identified as a circulating biomarker for cardiac events in patients with suspected acute coronary syndrome. At the molecular level, it seems that hypoxia acts as a modulator of SELENBP1, therefore reducing the oxidative stress and controlling the lower oxygen supply (46).

Previous studies have shown that circulating Se plays an important role in the pathogenesis of abnormal glucose metabolism, especially at high concentrations (47, 48). High exposure to Se can affect the expression of the main regulators of glycolysis and gluconeogenesis, through actions mediated by the GPX1 (49), as shown in studies that evidenced that the overexpression of this selenoprotein causes insulin resistance (50).

A review study has elucidated the relationship between Se status and cerebral Se homeostasis via SELENOP. In fact, SELENOP may be involved in some brain disorders, in particular in Alzheimer's disease, providing Se for brain tissue to produce selenoproteins. In addition, it competes with amyloid-β for metal ions and redox-active metals, such as copper and iron. This study points out the involvement of SELENOP in signaling pathways in neuronal and glial tissues, including neuronal calcium homeostasis and excitotoxicity (51).

## Selenium Supplementation

Brazil nuts (*Bertholletia excelsa, family Lecythidaceae*) are known to be the richest source of Se with high SeMet content and therefore, it has been widely used in studies of Se supplementation. Regular consumption of Brazil nuts results in optimum plasma Se and erythrocytes concentrations, as well as in better activity of selenoenzymes (52–55) antioxidant state (56), muscular retention (57), and inflammation status (54, 58). It is important to consider genetic variants in selenoprotein genes (55) and pre-stratification of the population prior to starting the trials as a way to avoid possible differentiated responses depending on the Se status in each individual (59). Studies on the effects of supplementation with Brazil-nuts on selenium biomarkers are shown in Table 1.

**Table 1:** Studies on the effects of supplementation with Brazil-nuts on selenium biomarkers.

**Studies**	**Country population**	**Supplemented dose (μg)**	**Ingestion period (w)**	**Se in plasma (μg/L)**		**SELENOP (ng/mL)**		**GPX (Ug/Hb) erythrocyte**	
				**Before**	**After**	**Before**	**After**	**Before**	**After**
Thomson et al. (52)	New Zealand 59 adult men	100	12	90.8 ± 12.63^a^	150.81 ± 17.4^a^			20.6 ± 4.4^a^,^b^	22.8 ± 5.0^a^
Cominetti et al. (53)	Brazil 37 obese women	290	8	55.7 ± 13.3^a^	132.5 ± 34.9^a^			36.6 ± 17.0^a^	53.6 ± 20.4^a^
Duarte et al. (54)	Brazil 55 obese women	1261.4	8	87.1 (82–97.7)^c^	244 (226–278)^c^	37.7 (16.1–51.9)^c^	55.5(37.1–150.6)^c^	48.7 (37.5–57.6)^c^	57.2 (45.8–67.5)^c^
Donadio et al. (55)	Brazil 130 healthy adults	300	8	90.7 (86.4–95.2)^d^	267.0 (252.8–282.0)^d^	3.4 (3.2–3.5)^d^mg/L	3.9 (3.7–4.1)^d^	61.8 (58.8–65.1)^d^	61.3 (57.7–65.1)^d^

a *Mean ± SD*.

b *Whole blood GPx was assayed as a measure of erythrocyte GPx activity*.

c *Median (interquartile interval)*.

d *Geometric means (CI 95%)*.

The effect of Brazil nuts on the human intestinal microbiota is still unknown. It is well-known, however, that Brazil nuts contain fiber, unsaturated fatty acids, and polyphenols that may impact the composition of the gut microbiota and overall gut health. A systematic review with meta-analysis including randomized controlled trials on nut consumption investigated the intake of almonds (*n* = 5 studies), walnuts (*n* = 3 studies), and pistachios (*n* = 1 study) and demonstrated a significant increase in the gut content of genera *Clostridium, Dialister, Lachnospira*, and *Roseburia*, as well as a significant decrease in *Parabacteroides*. The nuts did not show any significant influence on bacterial phyla, bacterial diversity or stool output (60). Other studies have only reported an increase in the abundance of butyrate-producing bacteria after nuts (61, 62) and pistachios (63) intake, without demonstrating any effect on the overall composition of the microbiome.

The Nutritional Prevention of Cancer (NPC) trial showed the effectiveness of supplementing 200 μg/day of Se, as selenized yeast, in reducing the risk of prostate, lung, and colorectal cancers (64). Se supplementation was also reported to decrease CVD and related mortalities (65, 66). In a group of healthy New Zealand men, Se supplementation as selenized yeast (Selplex, 200 μg/day) in the form of SeMet, significantly increased Se levels, improved the TXNDR activity and enhanced DNA stability (59). Due to the positive results from the NPC trial, the Selenium and Vitamin E Cancer Prevention Trial (SELECT) was undertaken in 35,000 healthy US men randomly assigned to 4 groups (selenium-−200 mcg/d from L-selenomethionine, vitamin E-−400 IU/d of all rac-alpha-tocopheryl acetate, selenium + vitamin E, and placebo). Neither selenium nor vitamin E, alone or in combination, was able to prevent prostate cancer in this population (67).

Observational studies and randomized clinical trials conducted with high-dose Se supplementation have shown controversy over the effects on diabetes mellitus, indicating that both excess and deficiency of Se may be associated with higher risks of diabetes mellitus type 2 (DM2) (68–71). In a NPC trial, participants were randomly assigned to receive 200 μg/day of Se (as high-selenium yeast). This group were more likely to develop DM2 than those assigned to placebo. In a randomized controlled study involving patients with DM2 and cardiovascular disease, supplementation with 200 μg Se/day resulted in a significant decrease in insulin, HOMA-IR, C-reactive protein and an increase in total antioxidant capacity (70).

## Selenium Toxicity

Se toxicity can affect individuals as a result of occasional overdose that usually occurs with intake of incorrectly formulated supplements (72) or due to the excess of Se intake in randomized clinical trials, in which doses of 200 μg/day or more are administered for a substantial period of time (73). Acute toxicity from excessive Se exposure causes stomach pain, headache, respiratory symptoms, changes in blood pressure, vomiting, and nausea. Chronic oral intake of high amounts of Se results in selenosis, a condition characterized by hair loss, deformation and loss of nails, tooth discoloration, garlic breath, gastrointestinal disturbances, skin rash, numbness, paralysis, and occasional hemiplegia (74). Other outcomes have been reported such as dermatitis, increased mortality (73), DM2 (68) and increased incidence of prostate cancer (67), which are also observed in Se deficiency.

Increased mortality has been reported at the highest dose of Se in the Danish PRECISE, a randomized, double-blinded, placebo-controlled, clinical trial performed with four groups treated with 100, 200, or 300 μg Se/day as Se-enriched yeast or placebo yeast. The results of this study warn that a 300-μg/day dose of Se (as Se yeast) taken for 5 years in a country with moderately low Se status can increase all-cause mortality by 10 years later (73).

The levels of dietary exposure that is able to induce selenosis and Se toxicity is difficult to establish due to the fact that toxicity is affected by the chemical form of Se and its bioavailability. Furthermore, interactions of Se with other dietary components, the individual's genotype and intestinal microbiota are also factors that influence the Se toxicity. Even in the face of Danish PRECISE, some populations exposed to excess Se did not develop adverse effects. Such conditions suggest that there are mechanisms for genetic adaptation that might be involved in oscillations in the Se intake, which are mediated by polymorphisms, complexation of SELENOP with toxic elements such as cadmium, arsenic, and mercury forming products of Se excretion (75, 76). The metabolism of Se by intestinal bacteria also favors the excretion of excess of Se (41, 77).

## Selenium and Gut Microbiota

The human digestive tract is inhabited by several microorganisms (bacteria, viruses, fungi, and protozoa) named microbiota, that includes ~100 trillion microorganisms (78). Bacterial cells are distributed unevenly along the gastrointestinal tract with more than 50 types of bacterial phylum. Only *Bacteroidetes* and *Firmicutes* are preserved in practically all individuals (79).

Human microbial colonization begins at birth and it is similar to the maternal vaginal microbiota. It is believed that intestinal colonization during birth and breastfeeding is essential to define the composition of the intestinal microbiota later in adulthood, although the determination of the composition of the microbiota is also influenced by several external and internal factors related to the host (80).

The microbiome is capable of encoding more than three million genes. It carries out a variety of metabolic functions not attainable by the human host, which includes the production of some types of vitamins and bioactive compounds, the synthesis of essential and non-essential amino acids, the metabolism of non-digestible carbohydrates and the activity in neural, hormonal, and immunological signaling through the gut-brain axis. Furthermore, it acts on the absorption of nutrients and as an epithelial barrier for pathogens (78). In this sense, imbalances in the intestinal ecosystem or in two-way communication with the brain are associated with gastrointestinal disorders, metabolic diseases, and neurobehavioral disorders. Therefore, strategies have been developed to manipulate the microbiome, with the aim of preventing and/or reversing conditions that are harmful to health (81).

Comparative genomics provides a powerful tool for investigating genes, pathways and evolutionary changes across multiple lineages (82). In the past decade, studies have been conducted evaluating the use of Sec Trait in ~600 bacterial and archaeal genomes, in which the organisms rich in selenoproteins were the anaerobic *Deltaproteobacteria* and *Clostridia* classes, especially *Syntrophobacter fumarroxidans*, with the highest prokaryotic selenoproteoma reported (83).

Traces of Se and related key genes have been evaluated in over 2,300 bacterial and archaea genomes, identifying a phylogenetic and genomic mosaic pattern among organisms using Se in different forms. This profile suggests new genes whose encoded proteins participate in Se metabolism and homeostasis in prokaryotes, such as YedE involved in Se transport, YedF which transcribes redox protein and LysR Se known as specific transcriptional Se-regulator (84).

Evolutionary trends in the use of Se and selenoproteins indicate more than 5,200 bacterial genomes, with the majority being related to the host, resulting in the largest Se utilization map in this realm. However, of this total, 2/3 of the bacteria do not use Se, suggesting that this ability has been lost over time. Environmental factors and use of Se were also investigated, revealing that Se-cofactor trait (68%) and Sec Trait (37%) appear to favor the conditions of host-associated bacteria, while SeU trait prefers aquatic species that have been isolated mainly from the sea or freshwater (85). These macro-evolutionary trends extend to cell respiration and temperature characteristics, in which anaerobic conditions can significantly promote the use of the Se-cofactor trait and lead to the evolution of new selenoprotein genes. Temperature seems to affect the use of Se, in which thermophilic (<40°C), mesophilic (20–40°C), and psychrophilic (<20°C) conditions favor the use of Sec Trait, Se-cofactor trait (mostly, but non-significant values) and SeU trait, respectively (85, 86). Thus, the human intestine can be a favorable ecosystem for the use of selenium by prokaryotes as the oxygen level in the colon, which is the site with the highest degree of Se absorption, is low and the optimum temperature varies between 25°C and 30°C (87).

### Dietary Factors and Intestinal Modulation

Genetic sequencing data carried out with 1,135 Dutch people detected 126 different environmental factors associated with microbiota, including diet, physical activity, diseases, and use of medicines (88). Specific foods and dietary patterns can influence the abundance of different types of bacteria in the intestine. For instance, the low intake of FODMAPs (Fermentable Oligosaccharides, Disaccharides, Monosaccharides, and Polyols) has been identified as a nutritional therapy indicated for the relief of gastrointestinal symptoms reported by patients with irritable bowel syndrome (IBS) and non-celiac sensitivity to gluten (89). Foods rich in fructans (wheat, rye, garlic, and onion) lactose (milk and dairy products), fructose (fruits and processed foods containing syrups), sorbitol, xylitol red fruits, and mushrooms are fermented by intestinal bacteria (*Actinobacteria*) and yeasts producing hydrogen and methane gases, resulting in bloating symptoms, abdominal pain, and diarrhea (90). In a meta-analysis study with randomized clinical trials, the low FODMAP diet was beneficial for remission of gastrointestinal symptoms in patients with IBS (91). However, the restriction of several foods may lead to a potential inadequacy of micronutrients in patients who follow this dietary recommendation, resulting in significant changes in the microbiota and metabolome, whose duration and clinical relevance are still unknown (92, 93).

### Selenium as a Modulating Agent of Intestinal Flora

Dietary Se influences both the host's selenium status and selenoproteoma expression. The intestinal microbiota can use the ingested Se for the expression of its own selenoproteins. Se affects the composition and colonization of the gut microbiota, which may interfere with the diversity of the microbiota and cause unique effects on microbial composition. About 1/4 of all bacteria have genes that encode selenoproteins. Some of them, such as *Escherichia coli, Clostridia*, and *Enterobacteria* classes, are able to colonize the gastrointestinal tract of humans and animals (94). Selenocysteine synthase (SelA) is a pyridoxal phosphate-dependent enzyme (PLP) (95) which catalyzes the formation of selenocysteinyl-tRNA in bacteria from a UGA decoding tRNASec (SelC) loaded with serine and selenophosphate, the product of the enzyme selenophosphate synthetase (SelD). Along with SelB, a specific translation factor of selenocysteinyl-tRNA, SelA, SelC, and SelD are components of bacterial Sec decoding, allowing the incorporation of Sec into specific UGA codons followed by a sequence of insertion of Sec elements (SECIS) (96).

The composition of the microbiota can also be modulated by metals that participate in microbial growth through respiratory mechanisms, as a source of energy for autotrophic growth, as well as to transfer and storage of electrons between cells (86). Manganese, zinc, selenium, and iron act as critical cofactors for bacterial enzymes responsible for DNA replication and transcription, antioxidant action, and cellular respiration (97). Iron and zinc are the metals used by almost all living organisms in metabolic and oxidation-reduction processes (98). Some species require Se for normal metabolic functions, for instance, *Escherichia coli* has three selenoproteins in its structure (99).

Selenocompounds are found in animal and plant sources with distinct bioavailability. In experimental models using rats, no differences in nutritional availability were observed between selenite, selenate, selenocyanate (SeCN), SeMeCys, SeMet, selenohomolanthionine (SeHLan), selenocenoine (SeCys2), 1β-methyl-acetyl-D-galactosamine (SeSug1), except for trimethylselenonium ion (TMSe) administered orally. The authors discussed these findings based on mechanisms related to gastrointestinal enzymes that can degrade bioselenocompounds into selenocompounds in the intestine (100).

Germ-free mice that were fed with diets with adequate and high Se levels modified their selenoproteoma expression in a similar way to that of the control group but showed higher levels and activity of GPX1 and methionine-R-sulfoxide reductase 1 (MSRB1) in the liver, suggesting partial sequestration of Se by intestinal microorganisms, therefore resulting in limited availability to the host. In these experiments, the genus *Parabacteroides* of the phylum *Bacteriodetes*, showed an opposite correlation with Se dietary supplementation. The study concluded that dietary Se affects both the composition of the gut microflora and the colonization of the gastrointestinal tract (99).

Zhai et al. (101) compared the effects of different levels of Se dietary supplements (deficient, adequate, and supranutritional) on the intestinal microbiota of mice. The animals' fecal microbiota transplantation was performed in one of the experiments. Supplementation conducted with different amounts of Se did not significantly alter the mice's intestinal microbiota. It rather induced significant changes in the composition of the gut microbiota. In comparison to the Se-deficient diet, supranutritional Se supplementation significantly decreased the abundance of *Dorea* sp. and increased the levels of microbes with potential protective effects against colitis and intestinal barrier dysfunction, such as *Turicibacter* and *Akkermansi. Dorea* sp. is one of the most common species of the intestinal microbiota that supplies hydrogen and carbon dioxide in the intestine. The authors concluded that Se supplementation can optimize the intestinal flora to protect against intestinal dysfunction.

### Microbiota as an Environment That Affects Selenium Status

**Figure 5:**
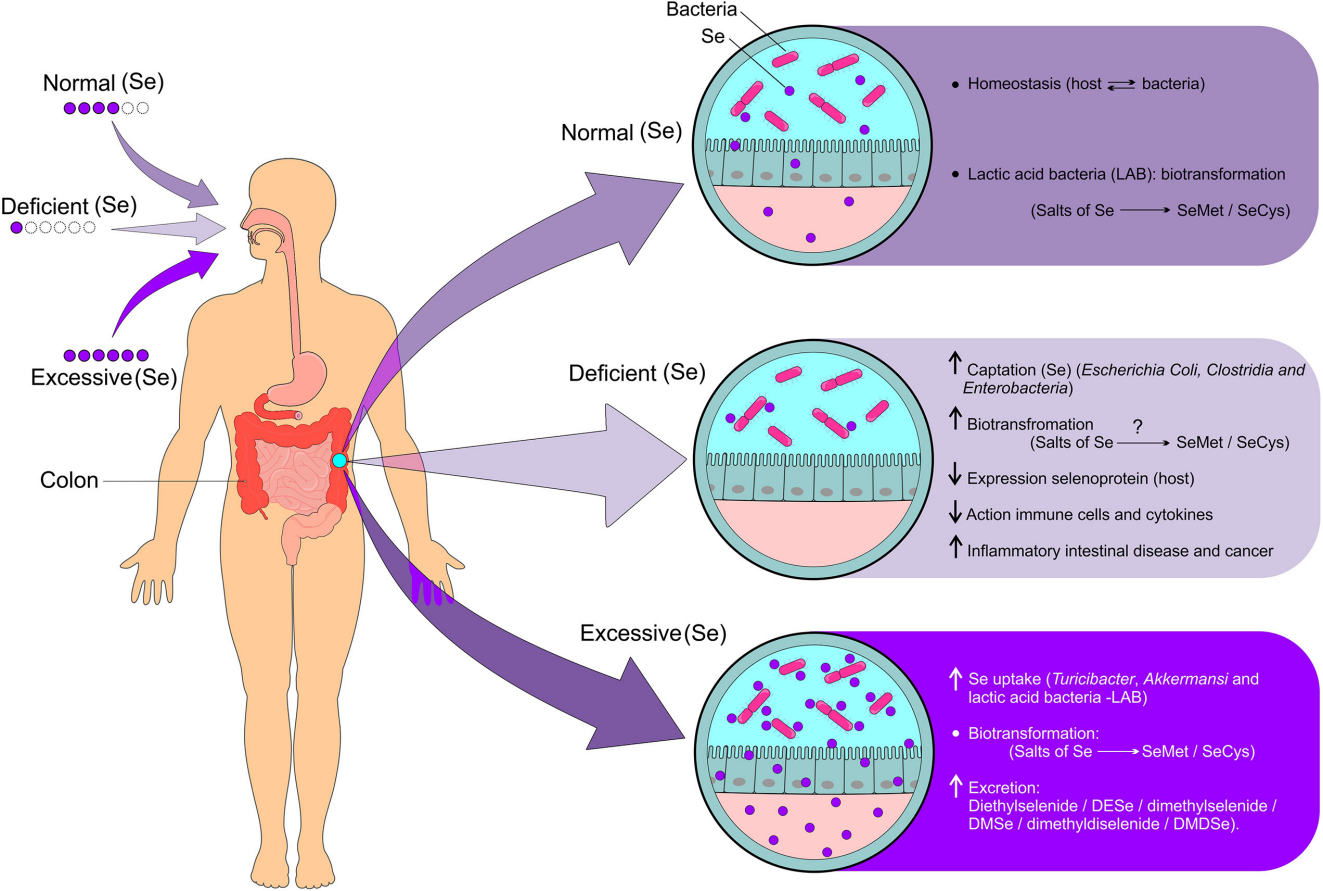
Modulation of the gut microbiota dependent on Se status and biotransformation of Se derivatives. Given the adequate intake of Se, homeostasis occurs due to the beneficial relationship between intestinal and host bacteria resulting in the biotransformation of Se compounds (Se salts metabolized into SeMet and SeCys). Se deficiency results in increased Se uptake by bacteria (*Escherichia coli, Clostridia*, and *Enterobacteria*), biotransformation of Se compounds (Se salts metabolized into SeMet and SeCys), decreased expression of selenoproteins by the host, decreased activation of Se immune cells, increased pro-inflammatory cytokines, and increased risk for IBD and cancer. On the other hand, excessive intake of Se causes increased uptake by bacteria such as *Turicibacter, Akkermansi*, and Lactic acid bacteria (LAB), biotransformation of Se compounds such as selenite (Se${\text{O}}_{3}^{2-}$) and selenate (Se${\text{O}}_{4}^{2-}$) which are metabolized into SeMet and SeCys, and increased excretion of volatile compounds from Se. Se, selenium; SeMet, selenomethionine; SeCys, selenocysteine; IBD, inflammatory bowel diseases (Figure illustration by Francisco Irochima Pinheiro).

Although the host and the intestinal microbiota mutually benefit from a symbiotic relationship, these environments can become competitors when the supply of micronutrients becomes limited. On the other hand, the intestinal microbiota favors the biotransformation of Se compounds, characterizing a dubious situation (Figure 5). The Se uptake by intestinal bacteria can negatively influence the expression of selenoproteins in the host, which results in a two to three times lower levels of selenoproteins under Se limiting conditions. The unfavorable consequences of this effect for humans and animals have not yet been evidenced. In view of the high propagated intake of probiotics, the metabolism of Se in these organisms should be investigated in order to assess whether a higher Se intake is recommended (94).

A study conducted with animal models indicated that the gut microbiota may affect the status of Se and the expression of selenoproteins. The colonization of germ-free (GF) mice has shown to induce the expression of the gastrointestinal form of several selenoproteins, even under conditions of Se-deficient diet. GF mice showed higher GPX and TXNRD1 activities in the intestine and liver, greater expression of GPX1 in the liver and GPX2 in the proximal and distal jejunum and colon, as well as greater activity of GPX1 and GPX2 in the colon. The study indicated that GF animals have less need for Se for selenoprotein biosynthesis than conventionally colonized animals. In addition, it has been observed that colonized animals have a higher risk for developing selenoprotein deficiency when the supply of Se becomes limited (94).

Another study has demonstrated that several inorganic and organic selenocompounds were metabolized to SeMet by the gut microflora of rats and that SeMet was incorporated into bacterial proteins. Proteins containing SeMet, available as a Se pool for the host animal, were accumulated in the gut microflora. The main urinary selenometabolite, SeSug1, was transformed into a nutritionally available selenocompound by the intestinal microflora. Finally, positive effects on the bioavailability of some bioselenocompounds, such as SeCN, MeSeCys, and SeSug1, were observed in the gut microflora (102).

Some bacterial species are able to benefit from Se by triggering some effects on bacterial pathogenesis. Faced with an infection by this type of bacteria, a complex interaction takes place between the host's immune response, the microbial pathogen, the microbiota, and the host's Se status. Bacteria that have Se-dependent enzymes can survive under anaerobic conditions in the mammalian gut. As a result, these bacteria benefit from the host by using Se to increase its virulence and pathogenicity (103).

Se deficiency can leave the individual immunocompromised, allowing the survival of bacteria that do not need Se to establish an infection and cause disease. The host's microbiota may also differ in the presence of Se, which can prevent infection by Se-dependent bacteria, either by competition for Se or by the production of toxic metabolites that can be harmful to pathogenic bacteria (103).

### Selenium, Microbiota, and Toxicity

The role of the intestinal microbiota in the excretion of SeMet and selenite has been investigated in rats. It has been reported that the excretion of excess of SeMet and selenite occurs through the production of methylated derivatives of Se and elemental Se from the biotransformation of L-selenomethionine and selenite (104). Another study corroborates this hypothesis by showing that the gut microflora of rats can metabolize L-SeMet to some metabolites (77).

Bacterial count and protein analysis have shown that the number of cells and protein concentrations in the cecum and colon suspensions of rats are similar, but the cecum microbiota of these animals may contain more metabolically active microorganisms for SeMet and selenite compared to those in the colon microbiota. Given the much larger relative size of the colon in humans, the metabolism of Se compounds in the human intestine is likely to occur mainly in the colon. The formation of these volatile compounds of methylated and elemental Se in the intestinal tract points to the role of the microbiota in protecting the host from toxicity due to high doses of Se supplements (104).

Significant increase in the absorption and distribution of cadmium and lead in the blood, gastrointestinal tract, kidneys, liver, and spleen were seen in germ-free mice exposed to cadmium or lead (5, 20, and 100 ppm) for 6 weeks in comparison to non-exposed animals. Thus, it seems that the microbiota act as a protective factor against heavy metals (105).

The role of Se has also been investigated against methylmercury (MeHg) poisoning though the modulation of gut flora and decomposition of this compound. Treatment with selenite for 90 days of rats poisoned with MeHg showed a modulation of flora abundance, specially *Bacteroidetes* and *Firmicutes* phyla. An increase in total mercury (THg) was found in fecal samples after treatment with Se on the 30th day. The percentage of MeHg in the poisoned group was between 81 and 105%, while 65–84% was found in the Se treated group, suggesting an increase in MeHg decomposition after treatment with Se (106).

### Selenium, Microbiota, and Diseases

Se and selenoproteins may play an important role in signaling pathways that are involved in the pathogenesis of some diseases, especially IBD (107), cancer (108), thyroid dysfunction (109), and neurogenerative disorders (110). The Se status may impact the expression of nuclear factor-κB (NF-κB) transcription factors and peroxisome proliferator activated receptor (PPAR) γ, which are involved in immune cell activation that ultimately results in various stages of inflammation (107). Thus, Se deficiency and inadequate selenoprotein expression impair innate and adaptive immune responses, especially at the colonic level where an increase in inflammatory cytokines is observed (25). In addition, low intake of Se might result in a phenotype of the gut microbiota that is more susceptible to colitis and infection by *Salmonella typhimurium*. On the other hand, a diet with sufficient or high levels of Se can optimize the gut microflora for protection against intestinal dysfunctions and chronic diseases (101).

#### Selenium, Microbiota, and Inflammatory Bowel Diseases

Crohn's disease and ulcerative colitis are IBD characterized by microbial dysbiosis that result in changes in intestinal motility and secretion, visceral hypersensitivity (hyperalgesia), and failure in the intestinal-brain communication (111). Se deficiency is common among patients with IBD, reaching 30.9% of cases (112). The importance of Se in improving IBD is attributed to the ability of the selenoproteins in reducing the inflammatory response (113, 114).

The nuclear factor erythroid factor 2-related factor 2 (Nrf2) also appears to contribute to redox homeostasis in epithelial cells (115). In a study conducted using an animal model of IBD, the lack of Nrf2 led to increased expression of inflammatory cytokines, such as TNFα and IL6 and increased expression of COX2 (116). Nrf2 can also stimulate the expression of TXNRD and GPX under adequate concentrations of Se (117). This relationship was explored in another study that found a positive association between plasma concentration of Se and the expression of Nrf2-related genes (118). In addition, a study showed that the lack of Nrf2 increases NF-?B activity, further intensifying oxidative stress (119).

Zhu et al. (120) investigated the protective effect of Se nanoparticles with *Ulva lactuca* polysaccharide (ULP-SeNPs) on DSS-induced acute colitis in mice. The main benefits were the reduction of CD68 in the colon, modulation of IL-6 and TNF-α, inactivation of macrophages and suppression of nuclear translocation of NF-κB.

Bacteria with pro-inflammatory activity, such as *Escherichia* and *Fusobacterium*, are increased in patients with IBD, whereas anti-inflammatory species such as *Faecalibacterium, Roseburia Clostridium coccoides, Clostridium leptum, prausnitzii*, and *Bifidobacterium* are reduced in this disease (121, 122). Other phyla of bacteria have been associated with the ingestion of Se in individuals with IBD. Dietary Se was positively correlated with the presence of *Firmicutes* and negatively correlated with *Verrucomicrobia* in patients with Crohn's disease and ulcerative colitis, respectively (123).

Animals treated with SeCys and selenocystine showed a reduction in the concentration of ROS and malondialdehyde (MDA), as well as an increase in intestinal activity of SOD and GPX, which seems to indicate a protective effect against damage to the gut mucosa. In addition, the levels of IL-1, MCP, IL-6, and TNF-α were significantly reduced in the group treated with SeCys (124).

Xu et al. (125) reported that the administration of Se nanoparticles with *Lactobacillus casei* ATCC 393 (*L. casei* 393-SeNPs) protected mice from intestinal barrier dysfunction and oxidative stress associated with enterotoxigenic *Escherichia coli* infection K88 (ETEC K88), when compared to the animals supplemented with *L. casei* alone. These findings suggest the ability of *L. casei* 393-SeNPs in maintaining intestinal epithelial integrity.

#### Selenium, Microbiota, and Cancer

The specific link between gut microbiota, selenium status, and cancer is difficult to establish, and multiple mechanisms may be involved in the complex interplay between microbiome, diet, and human host. It has been demonstrated that dietary Se affects both composition of the intestinal microbiota and colonization of the gastrointestinal tract, which, in turn, influence the host Se status and selenoproteome expression (99). The effect of the gut microbiota on selenoproteins and other molecules linked to redox homeostasis and those linked to the WNT/β-catenin signaling pathway may have an impact on the regulation of oxidative stress, apoptosis, inflammation, and immune response, suggesting a direct influence on increased risk of cancer (108).

Considering that Se uptake by intestinal microbiota occurs in conditions of imbalance, it might negatively impact the supply of Se to the host, therefore predisposing to cancer and gut dysfunctions. Deficiency of selenoproteins and molecules linked to redox homeostasis can lead to a gut microbiota phenotype that is more vulnerable to colitis, pathogen infections, and cancer (101). Lower expression of different selenoproteins have been described in colorectal adenomas and cancer tissues, while higher SELENOP concentrations were inversely associated with colorectal cancer risk (126).

Bacteria of the *Dorea* sp. genus, one of the most common species of the gut microbiota, are increased in conditions of deficiency of Se (101) and are associated with IBS, cancer, multiple sclerosis, and non-alcoholic liver disease (101, 127–130). Se deficiency and inadequate selenoprotein expression impairs innate and adaptive immune responses with higher levels of inflammatory cytokines, especially at colonic level. The effect of the gut microbiota on selenoproteins and other molecules linked to the redox homeostasis may have an impact in the regulation of oxidative stress, apoptosis, inflammation, and immune response, which appears to have a direct influence on cancer risk and development (108, 131).

On the other hand, the administration of probiotics enriched with organic Se seems to by a promising alternative for elimination of pathogenic bacteria in the case of IBD and colon cancer (132). Likewise, Porto et al. (133) showed that oral administration of *Saccharomyces cerevisiae* enriched with Se reduced eosinophil peroxidase activity, histopathological tissue damage and oxidative stress (lipid peroxidation and nitrite production) in the small intestine of mice. Therefore, clinical studies involving the biological function and bioaccessibility/bioavailability/bioactivity of selenoproteins and selenometabolites in different functional foods enriched with Se and nutraceuticals are highly recommended in order to confirm the findings of preclinical studies.

#### Selenium, Microbiota, and Thyroid Dysfunctions

The thyroid gland contains the highest amount of Se per mg of tissue in the body. Several proteins involved in thyroid metabolism contain Se, namely GPX (type I and II), DIOs, and TXNRD. Resident microbes of the colon metabolize Se, which is not absorbed by the host in the upper gastrointestinal tract. Microbes influence thyroid levels by regulating iodine uptake, degradation, and enterohepatic cycling. In addition, some minerals play an important role on interactions between host and microbiota, particularly selenium, iron, and zinc (134).

Besides having beneficial effects on the activity of the immune system, a healthy gut microbiota positively influences the thyroid function. Although dysbiosis has been found in autoimmune thyroid diseases (AITDs), it has also been reported in patients with thyroid carcinoma, in which an increased number of carcinogenic and inflammatory bacterial strains were observed. In addition, the composition of the gut microbiota has a major influence on the availability of essential micronutrients for the thyroid gland such as Se and zinc, which are co-factors for deiodination reactions that convert thyroxine (T4) into triiodothyronine (T3). Deficiency of these minerals might result from restrictive or unbalanced diets at any stage of life, which leads to a decreased production of thyroid hormones (135, 136).

The microbiota influences the uptake of Se and may alter the availability of L-thyroxine and toxicity of propylthiouracil (PTU) (134). In case of normal levels of Se, the thyoredoxin reductase system and SH-Px protect the thyrocytes from the activity of peroxides, whereas the apoptotic response to H_2_O_2_ is increased with Se deficiency (136). For instance, the decrease in the levels of *Lactobacillus* can interfere with the formation of iodothyronine deiodinases (DIOs) and, consequently, might result in thyroid dysfunctions (109, 137).

Several species of *Lactobacillus* are able to keep sodium selenite intracellularly as SeCys and SeMet, thus providing a more bioavailable form of Se, whose absorption by human cells is usually poor in its inorganic form (138). Therefore, the decrease in the amount of *Lactobacillus* in patients with thyroid disease might impart the bioavailability of Se and its role in the transformation of activated thyroid hormone. In addition, Se protects against oxidative damage during the synthesis of other hormones (109).

In a cohort study, the relationship between the gut microbiome, thyroid cancer, and thyroid nodules was confirmed. Among the findings, a relative abundance of *Butyricimonas* (*p* < 0.001) and a significant lower amount of *Lactobacillus* (*p* < 0.001) was observed in the group with thyroid cancer and in the group of thyroid nodules, respectively (86). The authors point out to the fact that *Lactobacillus* is an important genus in the human intestine that is able to improve the concentration of various metals in human cells including Se.

In human and rats, it has been proven that large amounts of conjugated iodothyronines can be hydrolyzed in fecal suspension. The diversity and structure of gut microbiota may play several roles in regulating the drug-controlled thyroidal metabolism (139). Some studies have corroborated that thyroid disorders are the causal factor in the relationship with gut microbes. Other studies have demonstrated that bacteria might act as the motivating factor, as thyroid function may be impaired in patients with small intestinal bacterial overgrowth (140, 141). However, the causative role of Se deficiency, thyroid and gut microbiota has not been thoroughly ascertained yet and further clinical studies are highly recommended.

#### Selenium, Microbiota, and Cardiovascular Diseases

The metabolic potential of gut microbiota has been identified as a contributing factor for the development of CVD (142). The intestinal microbiota produces signaling molecules such as lipopolysaccharide (LPS) and peptidoglycans that interact with host mucosal surface cells, often through the pattern recognition receptors (PRR) (143). In addition, the gut microbiota interacts with the host through the trimethylamine (TMA)/trimethylamine-N-oxide (TMAO) and short-chain fatty acids routes as well as through other routes related to biliary acids. Some of these molecules have shown to functionally interact with ghrelin, leptin, glucagon-like peptide 1 (GLP-1), and peptide YY (PYY), and to stimulate the parasympathetic nervous system. Such activities impact the metabolic processes related to the development of risk factors for CVD (142).

TMAO has gained considerable attention as a potential promoter of atherosclerosis, cardiometabolic diseases, arterial hypertension, ischemic stroke, atrial fibrillation, heart failure, and acute myocardial infarction (142, 144). Mice supplemented with choline or TMAO showed increased risk of thrombosis, in contrast to germ-free mice under the same diet, suggesting that gut microbiota and specific dietary nutrients that enhance TMAO generation seems to modulate platelet function and thrombosis potential *in vivo* (145).

Phosphatidylcholine and L-carnitine are metabolized by the intestinal microbiota producing trimethylamine gas (TMA), being further metabolized to TMAO by the liver enzymes of the host (144). A variety of enzymes are involved in the production of TMA from dietary components (146). Glycine betaine reductase (GrdH) is an enzyme that requires Se and is responsible for the production of TMA from glycine betaine (147). However, the role of Se in the TMA-generating pathways remains to be elucidated.

The role of gut microbiota in the oxidative stress process occurs through the uric acid metabolism. Higher levels of *Escherichia coli* result in greater uric acid decomposition, whereas elevated serum uric acid levels in patients with coronary heart disease are related to gut microbiota dysfunction. High levels of serum uric acid increase the production of oxygen free radical and induce endothelial dysfunction (148). Circulating Se is inversely associated with acid uric levels, suggesting the role of selenium in regulating the intracellular redox status (149).

Patients with type 2 diabetes and coronary heart disease have shown reduced hs-CRP, fasting blood glucose, insulin levels, HOMA-IR and increased nitric oxide (NO), total antioxidant capacity (TAC), and glutation (GSH) after use of 200 μg/day of Se and 8 x 10^9^ CFU/day of *Lactobacillus acidophilus, Lactobacillus reuteri, Lactobacillus fermentum*, and *Bifidobacterium bifidum* (2 x 10^9^ CFU/g each) for 12 days (150).

The bioavailability of Se on *Enterococcus faecium* CCDM 922A (EF) and *Streptococcus thermophilus* CCDM 144 (ST) and their respective forms enriched with Se, SeEF, and SeST, improved their antioxidant status in animal models (151). Selenium works by blocking the activation of nuclear factor-kB through modulation of expression of selenoprotein genes and by inhibiting the production of reactive oxygen species (ROS) (152). Moreover, probiotic may reduce inflammatory factors and oxidative damage by producing short chain fatty acids in the gut and by decreasing the production of free radicals (153). Probiotic and Se co-supplementation in diabetic patients with coronary heart disease showed beneficial effects on indicators of metabolic profiles related to cardiovascular disease.

Despite multiple human clinical studies revealing associations between gut microbiota composition with the development of cardiovascular diseases, few studies have provided mechanistic or causal evidence of a direct role of Se in gut microbiota in this context.

#### Selenium, Microbiota, and Glycemic Disorders

A study has shown that mice fed with a high-fat diet presented high plasma concentrations of LPS, which is a gram-negative bacterial translocation marker that is strongly related to insulin resistance, obesity, and diabetes. However, such LPS-induced metabolic responses were not observed in CD14 mutant mice, suggesting that the LPS/CD14 system may define the intensity of insulin sensitivity and related diseases (154). In this context, the presence of *Bifidobacterium* was associated with lower concentrations of LPS in the intestine, which resulted in a lower incidence of metabolic diseases (155). In addition to reducing the systemic inflammatory response, *Bifidobacterium* reduces intestinal permeability in patients with DM2 (156).

The antidiabetic effects of *Bifidobacterium* were more responsive when administered together with Se. *Bifidobacterium* enriched with sodium selenite (*B. longum* DD98, Se-B) mitigated oral glucose tolerance in diabetic mice, suggesting increased insulin sensitivity and protection of pancreatic β cells. These effects were dose dependent indicating the importance of administering adequate doses for better effectiveness of *B. longum* DD98, Se-B (157). Wei et al. (69) also evaluated the combined supplementation of Se with microorganisms in diabetic C57BL/6 mice, reporting that treatment with aqueous extracts of selenium-enriched *Auricularia auricular* (AESA) relieved liver damage triggered by oxidative stress in mice with DM.

Other mechanism involved in the prevention and treatment of insulin resistance relates to the production of short-chain fatty acids (SCFAs), especially butyrate (158). Increased concentration of butyrate in DM2 mice supplemented with live multi-strain probiotics was able to reduce HbA1C levels, improving glucose tolerance and insulin resistance (159). In addition, the administration of Se nanoparticles (0.9 mg/kg) demonstrated an increase in butyrate and in the amounts of beneficial bacteria such as *Lactobacillus* and *Faecalibacterium* (160). High concentrations of butyric acid, acetic acid, and isobutyric acid were identified in the feces of mice after oral administration of *B. longum* DD98, Se-B (157).

The positive effects of butyrate on insulin seems to be associated with an increase in the levels of GLP-1, which in turn lowers blood glucose in patients with DM2 (161) demonstrated that the administration of probiotic, VSL # 3, prevented and treated obesity and diabetes in mice. The mechanism discussed involves the probiotic-gut flora-butyrate-GLP-1 axis which is capable of promoting enhanced metabolic efficiency. Considering that supplementation with *B. longum* DD98, Se-B also resulted in increased secretion of GLP-1 and protected β cells, it has been speculated whether Se acts on this axis as a modulator of the deleterious effects caused by DM (157).

#### Selenium, Microbiota, and Neurological Diseases

With the discovery that some bacteria species produce chemicals similar to hormones and monoaminal neurotransmitters in the intestine, the microbiota-intestine-brain axis became evident. This bidirectional interaction allows the brain to influence the gastrointestinal functions as well as the immune functions (162). Oral administration of heat-killed *Candida kefyr* decreased the severity of experimental autoimmune encephalomyelitis (EAE), significantly reduced Th17 cells and increased regulatory T dendritic cells (DC) and CD103+. In addition to changes in intestinal immunity, changes in the microbiome have been observed such as increase in *Lactobacillales* and decrease in *Bacteroides* contents (163). In another study, the administration of Lactobacillus reuteri DSM 17938 modulated the immune response in EAE, decreasing T_H_1/T_H_17 cells and cytokines IFN-γ/IL-17 (164).

Neurodegenerative disorders are characterized by an increase in the production of reactive oxygen species (ROS) and a decline in the blood-brain barrier function. Due to the antioxidant property of Se, some selenoproteins play a neuroprotective role (110). TXNRD, for instance, maintains the redox balance and protects the dopaminergic cells, which are prone to oxidative stress in the pathophysiology of Parkinson's disease (165).

Fraga-Silva et al. (166) showed that the administration of Saccharomyces cerevisiae enriched with Se (Selemax) reduced the prevalence of EAE, increased the number of CD103 + dendritic cells and reduced the intestinal inflammatory process compared to the administration of *Saccharomyces cerevisiae* alone. In addition, Selemax supplementation demonstrated a neuroprotective effect by increasing the expression of protein tau in the CNS. Tau is the main protein associated with the stability of neuronal microtubules along with the MAPs (MAP1 and MAP2) (166). Long-term dietary supplementation (3 months) with Se-enriched yeast (Se-yeast) in triple transgenic mouse model of Alzheimer disease (AD), significantly improved spatial learning, retention of neuronal memory and activity (167).

In a study using humans, probiotic containing *Lactobacillus* acidophilus, *Bifidobacterium bifidum* and *Bifidobacterium longum* (2 × 10 ^9^ UFC/day each) and selenium co-supplementation (200 μg/day, sodium selenite) administered in patients with AD resulted in improved cognitive function and enhanced metabolic profile (168).

## Discussion

Recently, the role of Se in gut health has attracted the interest of the scientific community. This review points out that Brazil nuts, cereals, meat, fish, seafood, milk, and nuts are the best sources of Se and that both deficiency and excess of this metal are related to the occurrence of some diseases. Studies using human and rodent have shown that different doses and sources of Se supplementation can modulate gut microbiota with a positive or negative impact on the host's health (99, 100, 169). The microbial absorption of Se in the large intestine of rats was estimated to be 40–46% of the whole oral dose of Se. In addition, dietetic SeMet increased both fermentation and SCFA production in rats (170). The role of Se in the gut microbiota needs to be better investigated in humans as most studies have been conducted in animal models.

Both the structure and composition of the gut microbiome are significantly affected by genetic and external factors. Among the external factors, dietary pattern is the one that most rapidly alters the gut microbiome in real time, having important role in human health and in the development of chronic diseases (171). Moreover, growth and aging results in physiological changes that modify the gut microbiota (172). The composition of the gut microbiota can also be modulated by metals, therefore requiring a variety of cellular processes such as the system of capture of metal ions by bacteria or high affinity transporters (86). Until now, no specific Se carrier has been identified (84).

A systematic review with meta-analysis including randomized controlled trials on nuts consumption demonstrated a significant increase in the gut content of *Clostridium, Dialister, Lachnospira*, and *Roseburia*, as well as a significant decrease in *Parabacteroides* (60). This finding suggests that high consumption of nuts (a rich source of Se) regulates gut microbiota and promotes the expression of selenoproteins. An *in vivo* study using mice as the experimental model reported that Se supplementation can optimize the gut microbiota for protection against intestinal dysfunction (101). However, randomized clinical trials are necessary in order to investigate the real impact of Se supplementation on the microbiota and selenoprotein synthesis due to the lack of high-level evidence in the scientific literature.

It has been reported that Se plays a key role in cellular and paracellular permeability, as well as in cellular redox balance and inflammatory cell infiltration (173). Consistent with this finding, Se-deficiency adversely affected the gut barrier function and induced disturbances in the intestinal and immune responses in mice (169). Such events has recently been implicated in several chronic diseases ranging from IBD, DM2, and CVD to cancer and thyroid dysfunctions (135, 136, 169, 174, 175).

The metabolic pathways of selenium biotransformation in gut microbiota remain unclear, even though some bioproducts from Se metabolizing organisms enriched with sodium selenite have been manufactured (137). The rationale behind the use of selenium and probiotic co-supplementation is based on the antioxidant and anti-inflammatory effects of this treatment as observed in the metabolic responses of animal models. For instance, it has been reported that a 4-week probiotic and selenium co-administration to mice under a high-fat diet led to a significant decrease in MDA levels (176). Se-enriched probiotics present themselves as a less toxic alternative to supplementation and have demonstrated a protective effect against liver damage in rats (177) and possible antioxidant, anti-inflammatory, and anti-apoptosis properties (178).

This current review has shown that the composition of the microbiota can be modulated by the dietary Se, in which it can influence both the Se status of the host and the expression of the selenoproteoma. In return, the organism provides the nutrients used by bacteria for energy production and maintenance of their metabolic pathways, therefore characterizing a symbiotic relationship. The gut microbiota can interact with Se for the expression of its own selenoproteins. In addition, some species of intestinal microorganisms can improve the bioavailability of Se and protect against its toxicity. One question that remains unanswered is what constitutes an optimal health-promoting microbiome.

Ultimately, determining the full landscape of host-microbiota interactions and Se status will enable advances in the development of bioproducts involving selenium metabolizing microorganisms, which seems to be a safe alternative for studies about Se supplementation.

## Author Contributions

LP coordinated the elaboration of the manuscript. RF, KS-E, RC, and LP developed the layout of the manuscript, collected literature, and wrote the manuscript. FP collaborated with the layout of the manuscript and drew all figures. EA translated the entire text to English. LP and EA edited the final version of the manuscript. RF assisted in the reference management. All authors participated in the analysis and interpretation of data as well as in writing the manuscript. All authors approved the submitted version.

## Conflict of Interest

The authors declare that the research was conducted in the absence of any commercial or financial relationships that could be construed as a potential conflict of interest.
